# UV Sterilization Effects and Osteoblast Proliferation on Amorphous Carbon Films Classified Based on Optical Constants

**DOI:** 10.3390/bioengineering9100505

**Published:** 2022-09-26

**Authors:** Kazuya Kanasugi, Keita Arimura, Ali Alanazi, Yasuharu Ohgoe, Yoshinobu Manome, Masanori Hiratsuka, Kenji Hirakuri

**Affiliations:** 1 Department of Electrical and Electronic Engineering, Faculty of Engineering, Tokyo Denki University, 5 Senju Asahi-Cho, Adachi-Ku, Tokyo 120-8551, Japan; 2 Applied Medical Sciences College, King Saud University, Riyadh 11451, Saudi Arabia; 3 Division of Electronic Engineering, Faculty of Science and Engineering, Tokyo Denki University, Ishizaka, Hatoyama 350-0394, Saitama, Japan; 4 Core Research Facilities, The Jikei University School of Medicine, 3-25-8, Nishi-Shinbashi, Minato-Ku, Tokyo 105-8461, Japan; 5 Nanotec Corporation, Nanotechno-Plaza, 4-6, Kashiwa-Inter-Minami, Kashiwa 277-0874, Chiba, Japan

**Keywords:** osteoblast proliferation, amorphous carbon films, optical constants, UV sterilization

## Abstract

Optical classification methods that distinguish amorphous carbon films into six types based on refractive index and extinction coefficient have garnered increasing attention. In this study, five types of amorphous carbon films were prepared on Si substrates using different plasma processes, including physical and chemical vapor deposition. The refractive index and extinction coefficient of the amorphous carbon films were measured using spectroscopic ellipsometry, and the samples were classified into five amorphous carbon types—amorphous, hydrogenated amorphous, tetrahedral amorphous, polymer-like, and graphite-like carbon—based on optical constants. Each amorphous carbon type was irradiated with 253.7 nm UV treatment; the structure and surface properties of each were investigated before and after UV treatment. No significant changes were observed in film structure nor surface oxidation after UV sterilization progressed at approximately the same level for all amorphous carbon types. Osteoblast proliferation associated with amorphous carbon types was evaluated in vitro. Graphite-like carbon, which has relatively high surface oxidation levels, was associated with higher osteoblast proliferation levels than the other carbon types. Our findings inform the selection of suitable amorphous carbon types based on optical constants for use in specific medical devices related to osteoblasts, such as artificial joints and dental implants.

## 1. Introduction

Carbon-based dry coating materials, including diamond-like carbon (DLC), is garnering increased attention owing to its appealing medical properties, such as antithrombogenicity, cell affinity, and antibacterial effects. Hence, they are being assessed for applications in various biomedical devices, including artificial joints and dental implants [1,2,3,4]. DLC film is a general term used for disordered carbon films primarily composed of sp^2^ or sp^3^ hybrid orbital carbon bonds and hydrogen bonds, with a structure that can be widely controlled using deposition methods and deposition conditions, including precursors [1,5,6,7]. Therefore, various amorphous carbon films with different film structures and characteristics exist. However, the selection of an appropriate deposition method and conditions remains difficult for ordinary dry coating users to ultimately acquire the amorphous carbon film of interest.

The structure models of amorphous carbon films have been previously discussed [1]. Hiratsuka et al. proposed an optical classification method using the optical constants of amorphous carbon films [8]. This classification method allows amorphous carbon films to be distinguished into six types with different characteristics (amorphous carbon [a-C], hydrogenated amorphous carbon [a-C:H], tetrahedral amorphous carbon [ta-C], hydrogenated tetrahedral amorphous carbon [ta-C:H], polymer-like carbon [PLC], and graphite-like carbon [GLC]) according to the n-k plot (λ = 550 nm) based on the refractive index (n) and extinction coefficient (k) obtained following spectroscopic ellipsometry (SE) analysis [8]. The structure and various properties of these six types of amorphous carbon films are currently being evaluated in detail [6,9,10].

In this study, we focused on the various amorphous carbon films distinguished by this optical taxonomy and investigated their biological responses [11,12]. In biomedical applications, the design of bio-interfaces that directly contact blood, cells, and proteins is particularly important; however, the optical constants obtained by SE analysis provide average values for the entire amorphous carbon film without providing surface-related information [11,12]. Therefore, when distinguishing between types of amorphous carbon based on differences in bio-responsiveness obtained from optical constants, it is necessary to accurately understand the relationship between optical constants and surface conditions as a bio-interface. In a previous study, we investigated the relationship between fibroblast proliferation and optical constants between four amorphous carbon types (a-C, a-C:H, PLC, and GLC) with different deposition methods and conditions and surface properties [11]. We then confirmed that a-C and GLC, which have relatively high extinction coefficients, have a higher C=O binding ratio than a-C:H and PLC, which promote cell proliferation [11]. However, biological responsiveness, including cell adhesion, proliferation, and differentiation, differ depending on the cell type [13,14]. In addition, previous studies have not adequately discussed the impact of 253.7 nm UV irradiation sterilization treatment prior to cell culture [7,11,13]. In addition to ultraviolet sterilization, high-pressure steam sterilization and electron-beam sterilization are other common methods of sterilization. Gotzmann et al. reported that the number of defects, such as pinholes in amorphous carbon films prepared by different deposition methods, increased after repeated high-pressure steam sterilization treatments. They also stated that electron-beam treatment of these amorphous carbon films causes surface oxidation, which promotes hydrophilicity and fibroblast proliferation [15]. Therefore, electron-beam treatment is considered to be an effective sterilization method for amorphous carbon films, but the equipment is relatively large and expensive. On the other hand, ultraviolet sterilization equipment requires a low-temperature process, is relatively inexpensive, is easy to handle, and is widely applied to a variety of applications [16]. This treatment effectively inactivates unwanted bacteria and viruses on the sample surface using a wavelength between 200 and 280 nm (germicidal UV), which is easily absorbed by DNA/RNA, and is performed in specific environments, such as on a clean bench [16,17,18]. Shi et al. reported that the UV irradiation of hydrogenated DLC films in a wet environment causes structural changes, including the surface oxidation of DLC, which affects tribological properties [19]. Therefore, it is possible that UV sterilization in the cell culture process affects the structure and surface properties of the targeted amorphous carbon films. It is, therefore, necessary to clarify the biological response of amorphous carbon films to various cell lines and assess the effects of UV sterilization treatment on the film structure and surface properties.

In this study, five types of amorphous carbon films were prepared on Si substrates using various plasma processes, including the physical vapor deposition (PVD) and chemical vapor deposition (CVD) methods. First, these amorphous carbon films were classified based on optical constants, and the changes in amorphous carbon film structure and surface properties induced by 253.7 nm UV sterilization treatment were investigated. The relationship between osteoblast proliferation and amorphous carbon film surface properties, was then evaluated.

## 2. Materials and Methods

### 2.1. Amorphous Carbon Film Deposition Conditions

**Figure 1:**
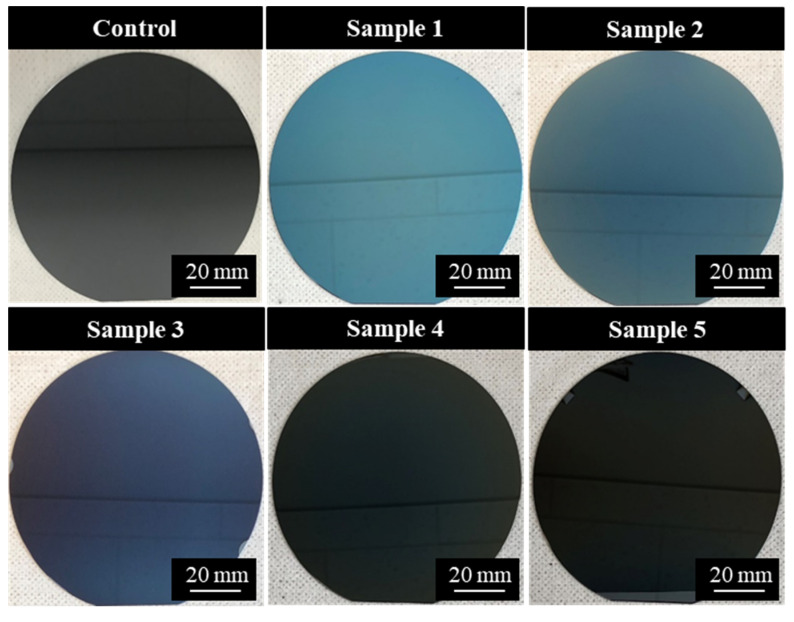
Amorphous carbon films deposited on Si and non-deposited Si substrate (control).

Amorphous carbon film deposition can be performed via PVD or CVD methods [1,6,20]. Those obtained through the PVD method have a relatively low hydrogen content and exhibit resistance to wear, abrasions, and corrosion [5,11]. Alternatively, the amorphous carbon films obtained through the CVD method have a relatively high hydrogen content and are highly flexible [5,21]. Although coating films have been investigated to improve biocompatibility for various biomaterials, their biological response is affected by surface roughness (especially micro-order). Therefore, the deposition of coating films on atomically flat Si substrates is preferred to minimize the influence of substrate surface roughness [22]. Accordingly, in this experiment, amorphous carbon films were deposited on 4-inch Si {100} substrates using various plasma processes, including PVD and CVD methods. The Si substrate has been used for the detailed characterization of the biological response and surface properties of amorphous carbon films [12,13,23,24]. The five types of amorphous carbon samples were cut into 10 mm × 10 mm squares for the cell culture test and surface analysis. The deposition conditions are listed in Table 1. Typical amorphous carbon film deposition methods, such as radio frequency plasma CVD (RF-PCVD), pulsed direct current plasma CVD (pulsed DC-PCVD), ionized evaporation, and high-power impulse magnetron sputtering (HiPIMS), were used. The precursor type, substrate bias voltage, and target voltage were arbitrarily adjusted to widely change the structure of the amorphous carbon film. In samples 4 and 5, hydrogen-free amorphous carbon films were prepared using the HiPIMS process with solid graphite targets [25]. Figure 1 illustrates the five types of amorphous carbon films deposited on Si substrates and an Si substrate (Control) on which no amorphous carbon film was deposited.

### 2.2. Spectroscopic Ellipsometer Analysis

The film thickness and optical constants of the amorphous carbon films deposited on the Si substrates were confirmed using a spectroscopic ellipsometer (Auto SE, HORIBA Ltd., Kyoto, Japan). The SE measurement resolution was 0.001 for optical constants and 1 Å for film thickness. The reflection amplitude ratio angle (ψ) and the phase difference (Δ) of s-polarized light and p-polarized light at a wavelength of 450–900 nm (photon energy: 1.4–2.8 eV) were measured using an SE with the angle of incidence fixed at 70°. Next, based on the SE spectrum, a regression analysis using a virtual thin-film multilayer model, assuming a surface layer/amorphous carbon layer/substrate, was performed, thereby reducing the chi-square (χ^2^) value and obtaining the refractive index (n) and extinction coefficient (k). Then, the types of amorphous carbon were classified according to the n-k plot at a wavelength of 550 nm, as defined in ISO23216:2021(E) [26].

### 2.3. Ultraviolet Sterilization Treatment

The 253.7 nm UV sterilization treatment (GL-15, Panasonic Ltd., Osaka, Japan) for each amorphous carbon film was irradiated for 1 h in a normal air atmosphere (temperature ~25 °C, humidity ~64%). The distance between the UV sterilization lamp and the sample was approximately 60 cm. The UV irradiance on the surface of the samples was measured using an illuminance meter (SP-82UV, Mothertool Co., Ltd., Nagano, Japan).

### 2.4. Structural Analysis

The structure of each amorphous carbon film before and after UV sterilization treatment at 253.7 nm for 1 h was confirmed using Raman spectroscopy (Raman: SpectraPro 2750, Princeton Instruments Inc., Acton, MA, USA). For the analysis, the laser output was 1 mW, laser wavelength was 532 nm, exposure time was 30 s, and number of integrations was set to 2. The Raman spectrum of the amorphous carbon films has a D peak (~1350 cm^−1^), due to the disordered structure, and a G peak (~1550 cm^−1^), due to the graphite structure, which has long been used to evaluate the film structure of amorphous carbon [1]. To characterize the film structure of each amorphous carbon type classified based on optical constants, these two peaks were waveform-separated by a Gaussian function, and their I_D_/I_G_ intensity ratio, G-peak position, and full width at half maximum (FWHM (G)) of G-peak were derived.

### 2.5. Surface Analysis

During surface analysis, the wettability, surface roughness, and surface composition of each amorphous carbon sample before and after UV sterilization treatment were evaluated. The wettability of the DLC film was judged from the static contact angle of pure water. For contact angle measurement, 2 μL of pure water was dropped onto the surface of the amorphous carbon film at room temperature (~20 °C). The pure water was membrane-filtered deionized water purified by ion exchange, and the contact angle was obtained using the θ/2 method [12]. Furthermore, in this contact angle evaluation, the drop position of the liquid was changed, and the evaluation was repeated ten times to account for measurement variation.

The surface roughness of the amorphous carbon films was evaluated using atomic force microscopy (AFM; SPM-9700HT, Shimadzu Ltd., Kyoto, Japan). The AFM analysis confirmed the root mean square roughness in the range of 10 μm × 10 μm in the contact mode (n = 3 or 4) [12].

The surface composition of amorphous carbon was analyzed using X-ray photoelectron spectroscopy (XPS; JPS-9000MC, JEOL Ltd., Tokyo, Japan). Carbon 1s (C1s) and oxygen 1s (O1s) peaks were analyzed using non-monochromatic radiation (MgKα source, 10 mA, 10 kV). The photoelectron extraction angle was set to 45° and the path energy to 10 eV. Although there is much debate regarding the correction procedure for XPS spectra, in this experiment, we used our previous protocol as a guide. That is, we standardized the C1s maximum peak value to 1 and shift-corrected it such that the C1s maximum peak occurred at 284.5 eV [12,27]. The C1s peak of each amorphous carbon sample obtained was waveform-separated into C-C sp^2^, C-C sp^3^, C-O, C=O, and O=C-O. All binding energies were calculated with reference to the carbon 1s peak of the surface at the C-C sp^2^ bond (~284.0 eV) [11,12,28].

### 2.6. Cell Culture Test

The cell proliferation properties of each amorphous carbon sample were evaluated through a cell culture test in vitro using mouse-derived osteoblasts (MC-3T3). The MC3T3 cells harvested from C57BL/6 mouse calvaria were obtained from the American Type Culture Collection (ATCC, Manassas, VA, USA). MC-3T3 cells are widely used for evaluating the biological responsiveness of amorphous carbon films [12,13,29]. Prior to cell culture, all amorphous carbon samples were subjected to 253.7 nm UV sterilization for 1 h. The UV sterilization treatment time was selected based on previous studies [11,12,30]. Next, each sample (n = 6) was cut into 10 mm × 10 mm squares and placed into a 12-well cell culture plate, and MC-3T3 cells were cultured on the amorphous carbon film for 72 h. Table 2 shows the cell culture conditions [12].

After cell culture, the living cells adhered to the amorphous carbon surface were disassociated using trypsin–EDTA solution (0.25 *w*/*v*, trypsin 1 mmol/L EDTA·4 Na Solution with Phenol Red; Wako Ltd., Osaka, Japan). Next, the CellTiter-Blue^®^ viability assay (Promega, Madison, WI, USA) was used to evaluate the number of living cells. In this assay, resazurin, a redox dye, is converted to fluorescent resorufin by the live cells. The number of live cells was estimated by measuring (n = 5) the fluorescence emission intensity (wavelength: 580 nm) using a plate reader (2300 En Spire, Perkin Elmer Inc., Waltham, MA, USA). The cell proliferation rate for the Si substrate (control) was normalized to 1. The Si substrate is non-toxic to osteoblasts and is associated with good osteoclast affinity [30]. A ratio near “1” indicates that cell proliferation is similar to that observed under control conditions. Alternatively, values higher than “1” indicate increased cell proliferation over the control.

Following amorphous carbon film lyophilization, the cell coating state on the amorphous carbon film was observed after 72 h using scanning electron microscopy (Regulus 8100, Tokyo, HITACHI Ltd., Kyoto, Japan) [12].

### 2.7. Statistical Analysis

To confirm whether the differences between amorphous carbon samples were significant, a Tukey’s multiple comparison test (sample number: 30; n = 6 × 5 cycles) was performed. In this test, differences associated with a *p*-value < 0.05 were considered significant; that is, if *p* < 0.05, there was a significant difference in cell proliferation between samples [12].

## 3. Results and Discussion

### 3.1. Classification of Amorphous Carbon Films Based on Optical Constants

Film thickness, refractive index, and the extinction coefficient of the five types of amorphous carbon films prepared using various deposition methods were evaluated by SE analysis (Table 3). In the SE regression analysis, χ^2^ became sufficiently small to conclude appropriate fitting. The thickness of the amorphous carbon films was controlled to 90–175 nm. In a previous study, it was reported that the cell growth of PLC films deposited on Si substrates by a radio frequency plasma CVD process is accelerated when the film thickness reaches about 300 nm [12]. Furthermore, in that report, no significant difference in the surface composition of the PLC film due to its thickness (film growth) was observed, and it was considered that the roughening of the surface on a nano-order scale affected cell growth [12]. Therefore, in this experiment, the deposition conditions were adjusted so that the thickness of each amorphous carbon film was at least <300 nm and thus the effect of surface roughness would not increase with increasing film thickness. The optical constant (λ = 550 nm) of the amorphous carbon films changed depending on the deposition method and conditions, with n between 1.870 and 2.500 and k between 0.040 and 0.690. Based on the n-k plot, our amorphous carbon samples were classified into five amorphous carbon types (a-C, a-C:H, ta-C, PLC, and GLC) [26]. Of these five types, three are considered DLC: a-C, a-C:H, and ta-C [6,31,32]. Generally, amorphous carbon types, such as GLC and a-C have high sp^2^ content and low hydrogen content [6,9,31]. In addition, PLC and a-C:H are characterized by high hydrogen content, leading to a decrease in the extinction coefficient and the number of π–π* bonds within the sp^2^ sites [6,9,31]. Amorphous carbon types with a relatively high refractive index, such as ta-C:H, exhibit high sp^3^ content and low hydrogen content [33]. That is, it can be inferred that the structures of the five amorphous carbon types classified based on the optical constants differ from each other.

### 3.2. Structural Changes in Amorphous Carbon Films Following UV Sterilization Treatment

**Figure 2:**
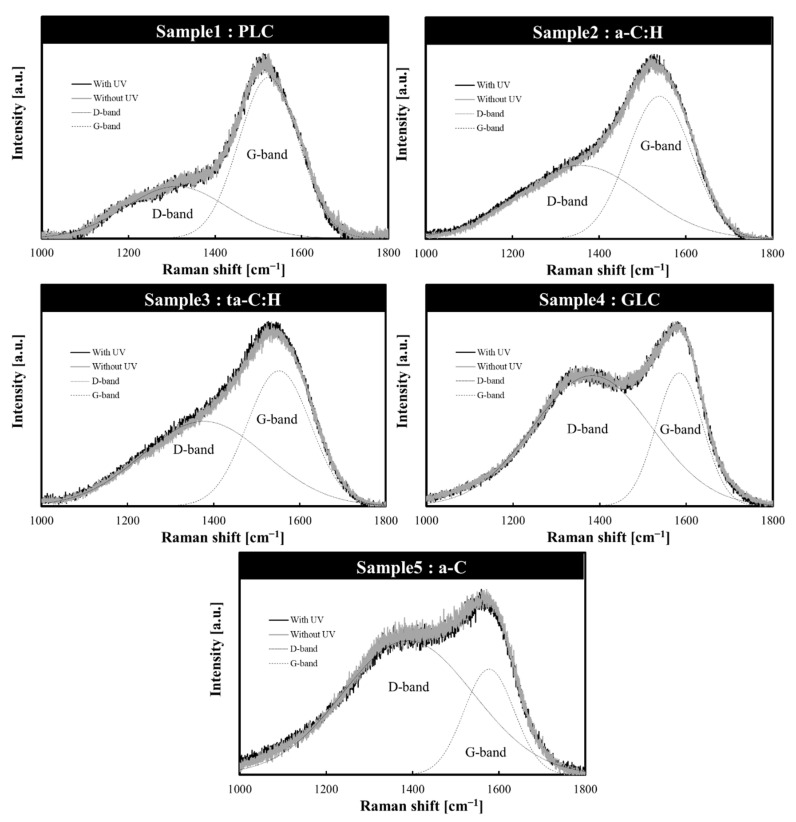
Amorphous carbon film structures classified based on optical constants (before and after UV sterilization). PLC, polymer-like carbon; a-C, amorphous carbon; a-C:H, hydrogenated amorphous carbon; ta-C:H, hydrogenated tetrahedral amorphous carbon; GLC, graphite-like carbon.

The structures of the five amorphous carbon films before and after UV sterilization treatment were confirmed using Raman spectroscopy. The Gaussian fitting results of the Raman spectrum are shown in Table 4, and the Raman spectrum of each sample is shown in Figure 2. In all five samples, the G and D-peaks characteristics of amorphous carbon were detected; the two bands were broad and overlapped, suggesting that they have a disordered amorphous carbon structure [1,34]. We also observed that GLC and a-C, which have relatively high extinction coefficients among the analyzed amorphous carbon types, had a large I_D_/I_G_ intensity ratio, a G-peak position at a relatively high wavenumber, and a small FWHM. In the case of visible light excitation, the I_D_/I_G_ intensity ratio depends on the size and number of sp^2^ clusters in the amorphous carbon film [1,35]. The G-peak position shifts to the lower wavenumber side and the FWHM (G) increases with increasing hydrogen content and sp^3^/sp^2^ ratio [1,6,25]. This suggests that GLC and a-C, which have relatively high extinction coefficients, present numerous sp^2^ bonds and low hydrogen content, as expected. The GLC type with highest harmonic side G-peak position and lowest FWHM (G) is expected to have more sp^2^ bonds than the other amorphous carbon types. These Raman spectroscopic findings confirm that the structures of the five amorphous carbon types classified based on the optical constants differ from each other. 

No significant difference was observed when comparing the Raman spectra of each amorphous carbon sample before and after UV sterilization. Since these Raman spectra were averaged over the entire film, it was possible that they did not capture the slight structural changes in the surface layer of the amorphous carbon film. In other words, it can be inferred that the 253.7 nm UV integrated exposure of this experiment would not significantly affect the bulk structure of amorphous carbon. It should be noted that the UV dose on the surface of the amorphous carbon sample was 6 μW/cm^2^, which is a sufficient UV integrated dose (21,600 μW × s/cm^2^) to sterilize 99% of bacteria [16]. 

### 3.3. Changes in Amorphous Carbon Film Surface Properties Following UV Sterilization Treatment

The wettability, surface roughness, and surface composition of amorphous carbon films before and after UV sterilization were evaluated. The water contact angles of the five amorphous carbon samples were within the measured error range, with no evident differences between the film types (Table 5). Similarly, no change in water contact angle was observed for each amorphous carbon type before and after UV sterilization. Although the surface roughness of each amorphous carbon sample increased slightly compared to that observed for the control, the difference between samples was in the order of nanometers (<3 nm), which was not significant. Hence, it was demonstrated that for both film types, at least a film thickness of <300 nm would not result in a significant difference in surface roughness. Similarly, the surface roughness of each amorphous carbon sample before and after UV sterilization was also within the measurement error. In other words, in this experiment, the effect of the roughness on cell growth is considered to be very small.

**Figure 3:**
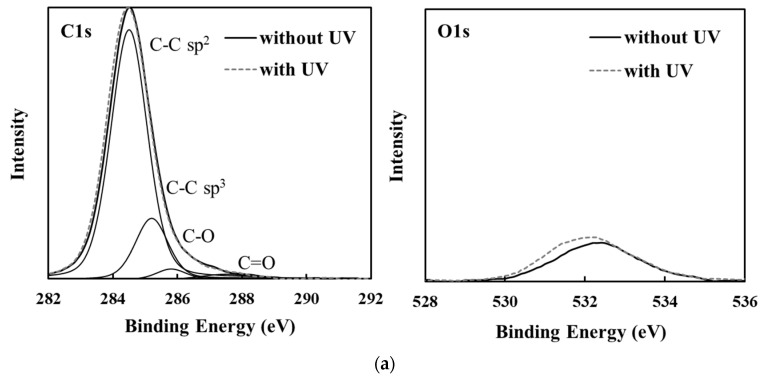

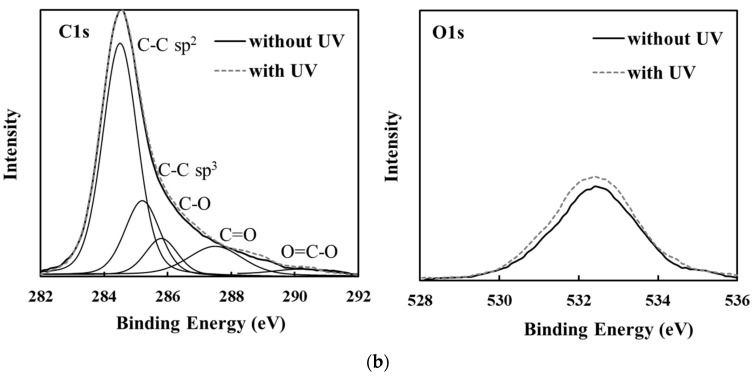
XPS spectra of amorphous carbon films before and after UV sterilization treatment. (**a**) Lower type and (**b**) higher type of oxygen functional group content.

XPS analysis was performed to confirm the surface composition of each amorphous carbon sample. The relatively high and low intensity of oxygen functional groups among the amorphous carbon types are apparent in the representative XPS analysis results in Figure 3. The C1s peaks were resolved into C-C sp^2^, C-C sp^3^, C-O, C=O, and O=C-O [11,28]. Table 6 shows the O1s/C1s ratio obtained using XPS analysis and the waveform separation area of the C1s spectra.

**Figure 4:**
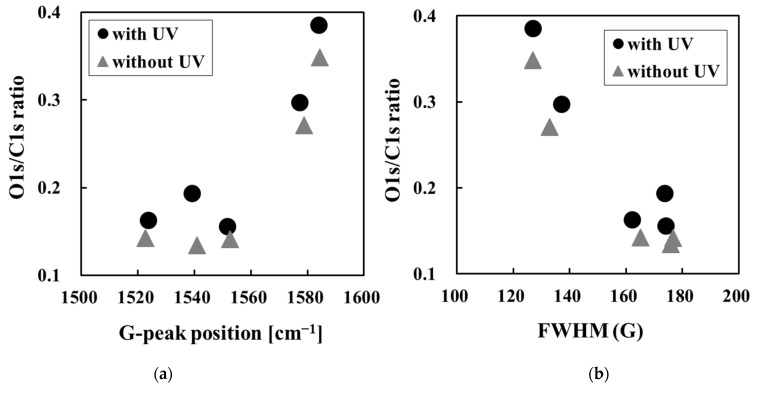
Correlation between O1s/C1s ratio and structure on amorphous carbon films before and after UV sterilization treatment. (**a**) G-peak position and (**b**) FWHM (G) of Raman spectra.

The GLC types had higher O1s/C1s ratios than the other types with or without UV sterilization, and oxygen functional groups such as C-O, C=O, and O=C-O were strongly detected. Hydrogen-terminated amorphous carbon surfaces, such as C-H bonds, inhibit the introduction of functional groups by surface oxidation from air and humidity [11,36,37]. C–C sp^2^ bonds with π bonds are more reactive than C–C sp^3^ bonds because π electrons are widely scattered [11,37,38]. That is, the surface oxidation of amorphous carbon films is considered to progress as the sp^2^ bond ratio increases, without hydrogen termination. Therefore, GLC has a relatively high O1s/C1s ratio and oxygen functional groups. Figure 4 shows the correlation between O1s/C1s ratio and G-peak position of the amorphous carbon films. The higher the O1s/C1s ratio, the higher the G peak position and the smaller the FWHM (G). As mentioned earlier, the G peak position and FWHM (G) affect the sp^2^ bonding of amorphous carbon films. Hence, the O1s/C1s ratio is higher due to the increase in sp^2^ bonds in amorphous carbon [1,6,25].

When focusing on the O1s/C1s ratio of each amorphous carbon film before and after UV sterilization, the O1s/C1s ratio was slightly higher for all DLC types after UV sterilization treatment, although the order of the ratios remained constant. Specifically, at the UV integrated exposure of 21,600 μW × s/cm^2^ in this experiment, the incremental O1s/C1s ratio of each sample before and after UV sterilization treatment was in the range of 0.2–0.6, and the average water contact angle became hydrophilic in the range of 1–6°. The photon energy of the 253.7 nm UV sterilization used in this experiment was 112 kcal/mol, which is higher than the average bond dissociation energy of a C-H (97.6 kcal/mol) or C-C bond (84.3 kcal/mol) [19,39]. In addition, during UV treatment in air, the oxygen generates active species, such as ozone and atomic oxygen, by UV light [19]. Therefore, during the UV sterilization process, bonds present on the amorphous carbon surface are likely dissociated, while highly polar oxygen functional groups, such as hydroxyl groups (-OH) and carboxyl groups (-COOH) are produced by the reaction of moisture and oxygen in the atmosphere and with reactive species [19,37,40]. Furthermore, no significant surface properties or structural changes of the amorphous carbon film were observed in this experiment, as in previous studies [19,40]. This may be due to differences in the amount of UV irradiation and the amount of integrated exposure to the sample surface. It has also been reported that different UV irradiation atmospheres, such as vacuums, affect the treatment effect [19,40]. These results highlight the necessity to carefully examine and manage the effects of the UV sterilization treatment condition on surface composition and structure when an amorphous carbon film is used as a bio-interface in the medical field where UV sterilization is required.

### 3.4. Classification of Amorphous Carbon for Osteoblast Proliferation Based on Optical Constants

The osteoblast proliferation associated with the five types of amorphous carbon films subjected to UV sterilization and that of the Si substrate (control) were evaluated using an in vitro cell culture (Table 7). 

**Figure 5:**
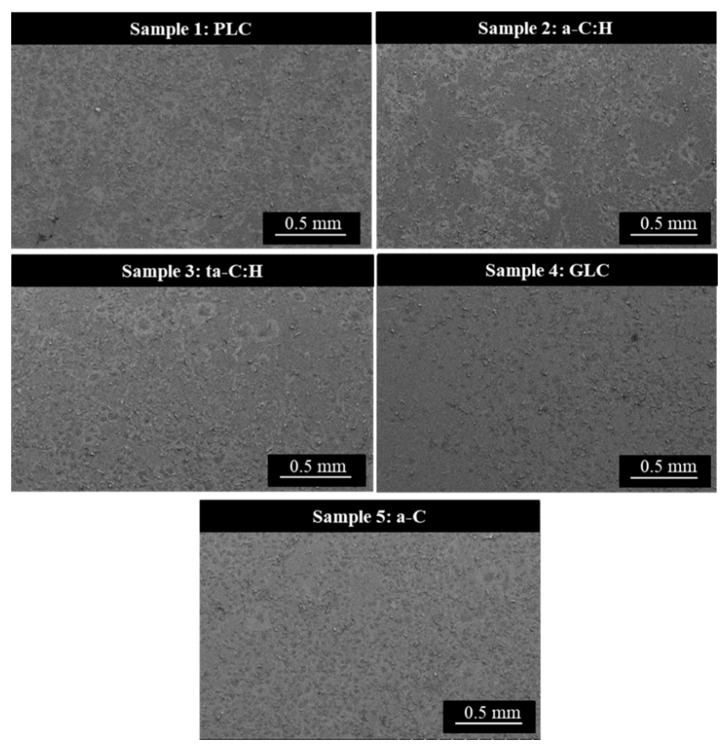
Images of cells cultured for 72 h on amorphous carbon samples. PLC, polymer-like carbon; a-C, amorphous carbon; a-C:H, hydrogenated amorphous carbon; ta-C:H, hydrogenated tetrahedral amorphous carbon; GLC, graphite-like carbon.

During the cell culture test, all amorphous carbon films were found to be stable in solution without peeling off from the Si substrate. The osteoblasts were then cultured on the amorphous carbon film for 72 h, and a thick layer of osteoblasts was observed on all samples. (Figure 5). Moreover, since fluorescence emission from living cells was detected, the five amorphous carbon samples were considered non-toxic to osteoblasts and exhibited a good osteoblast affinity. The cell proliferation levels of the five amorphous carbon film types were classified into two groups equal to or higher than the cell proliferation levels observed in the control group. The GLC type among amorphous carbon types had a high osteoblast proliferation level.

**Figure 6:**
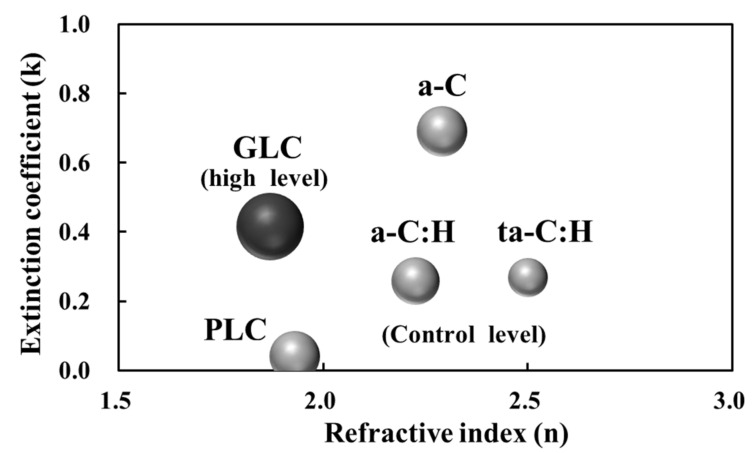
Amorphous carbon classification based on osteoblast proliferation and optical constants. The size of spheres represents osteoblast proliferation associated with each amorphous carbon type. The difference in the level of Si substrate equivalence (control) or higher is expressed using color coding. PLC, polymer-like carbon; a-C, amorphous carbon; a-C:H, hydrogenated amorphous carbon; ta-C:H, hydrogenated tetrahedral amorphous carbon; GLC, graphite-like carbon.

Figure 6 shows the classification of amorphous carbon for osteoblast proliferation based on optical constants. Amorphous carbon films were divided into two types: GLC, which has a relatively high extinction coefficient and low refractive index, and PLC, a-C:H, ta-C:H, and a-C (other amorphous carbon types).

**Figure 7:**
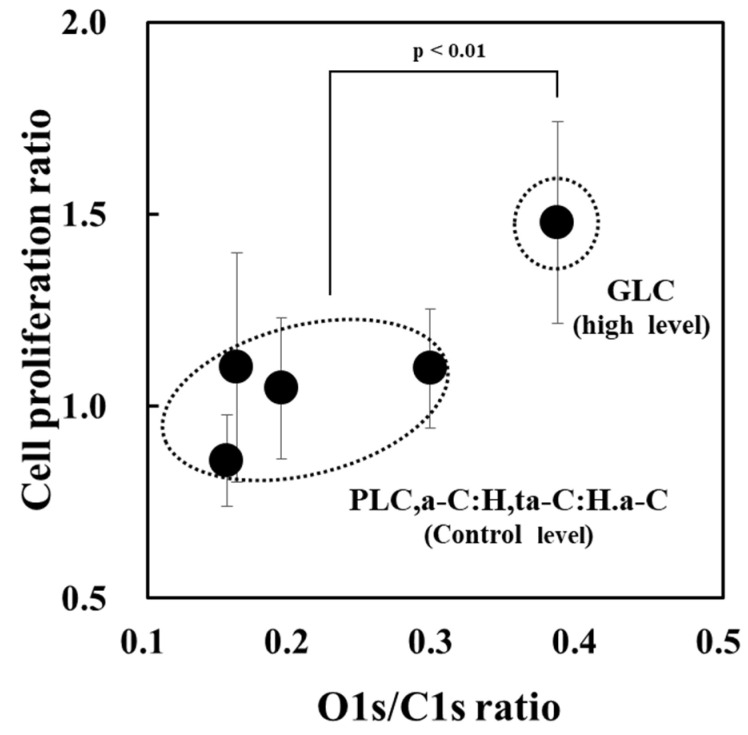
O1s/C1s and osteoblast proliferation on amorphous carbon films subjected to UV sterilization. PLC, polymer-like carbon; a-C, amorphous carbon; a-C:H, hydrogenated amorphous carbon; ta-C:H, hydrogenated tetrahedral amorphous carbon; GLC, graphite-like carbon.

Figure 7 shows the relationship between the O1s/C1s ratio and osteoblast proliferation of amorphous carbon films subjected to the UV sterilization treatment. The results show that the GLC type, which exhibits high osteoblast proliferation levels, had a higher O1s/C1s ratio than the other amorphous carbon types. This enhanced GLC oxidation may be due to its lower hydrogen content and higher ratio of highly reactive sp^2^ bonds compared to other amorphous carbons, including DLC. As a supplement, the GLC type proposed by Japan Diamond New Forum refers to those with hydrogen content <5 atm% and sp^3^/(sp^2^+sp^3^) ratio < 20% and that these ratios are the lowest compared to ratios of other film types [31,41]. Therefore, we believe it is reasonable that the GLC type has a relatively higher O1s/C1s ratio and more oxygen functional groups than other types of films, including a-C. Liu et al. reported that amorphous carbon films with low hydrogen content have more unpaired electrons on the surface that form covalent bonds with adsorbed proteins, thus maintaining the function of the adsorbed proteins and improving osteoblast affinity [29]. Moreover, the generation of oxygen functional groups, such as C-O, C=O, and O=C-O, on the amorphous carbon surface increases the zeta potential and affects cell proliferation [11,28]. That is, osteoblast proliferation is promoted in GLC, which has a low hydrogen content and relatively high O1s/C1s ratio. However, it has been reported that amorphous carbon films prepared by HiPIMS undergo surface oxidation as the film thickness increases [42]. Therefore, with a focus on the GLC type, it is necessary to further investigate the relationship between its film thickness, surface composition conditions, and cell proliferation. Furthermore, osteoblast proliferation between amorphous carbons other than GLC with different film structures and surface oxidation levels was within the range of variation of the experimental system with no significant differences observed. However, differences may be observed by changing variables, such as cell culture period and sample size. Additionally, although the influence of various GLC deposition processes, including HiPIMS, requires further investigation, based on the present results and previous reports, PVD processes using solid graphite feedstock, such as HiPIMS and unbalanced magnetron sputtering, seem to be effective for GLC fabrication as defined by the optical classification method [6,8,9,11,12].

## 4. Conclusions

In this study, five types of amorphous carbon films were prepared on Si substrates using various deposition methods, including the CVD and PVD methods. The refractive index and extinction coefficient of the amorphous carbon films were measured using SE, and the samples were classified into five amorphous carbon types: a-C, a-C:H, ta-C:H, PLC, and GLC. The effects of UV sterilization treatment at 253.7 nm on these amorphous carbon films and their osteoblast proliferative properties were then investigated. The UV irradiation of these films for 1 h did not significantly alter their film structure; however, the O1s/C1s ratio for all amorphous carbon types was slightly higher following irradiation. Next, we demonstrated that these amorphous carbon types were non-toxic to osteoblasts and exhibited good cell proliferative properties. Furthermore, we discovered that GLC exhibits a higher osteoblast proliferation level than the other amorphous carbon types, which might be explained by the relatively high surface oxidation levels of the GLC type.

The influence of other deposition methods, UV sterilization condition (treatment time and atmosphere), and substrate types not tested in this experiment will be examined in the future. Nonetheless, we consider that our results will guide industry amorphous carbon users in their selection of desired amorphous carbon types from the optical constants when using amorphous carbon films in certain medical devices involving osteoblasts, such as artificial joints and dental implants.

## Figures and Tables

**Table 1:** Amorphous carbon film deposition conditions.

Sample No.	Equipment	Deposition Method		Precursor	Gas Pressure (Pa)	Substrate Bias Voltage (kV)	Target Voltage (kV)
1	ISDD4, Nanotec Co., Chiba, Japan	CVD	RF-PCVD	Hydrogenated gas (C_6_H_6_: 13 sccm)	0.46	-	-
2	NPS330, Nanotec Co., Chiba, Japan		Pulsed DC-PCVD	Hydrogenated gas (C_6_H_6_: 20 sccm)	1.70	1.5	-
3		PVD	Ionized evaporation	Hydrogenated gas (C_6_H_6_: 1.4 sccm)	0.12	1.0	-
4			HiPIMS	Graphite solid target Ar gas (50 sccm)	0.82	0.0	0.75
5			HiPIMS	Graphite solid target Ar gas (40 sccm)	0.70	0.0	0.90

CVD, chemical vapor deposition; PVD, physical vapor deposition; RF-PCVD, radio frequency plasma CVD; Pulsed DC-PCVD, pulsed direct current plasma CVD; HiPIMS, high-power impulse magnetron sputtering.

**Table 2:** Cell culture conditions.

**Cell Line**	Osteoblasts (MC-3T3)
**Seeding density**	1.0 × 10^4^ cells/cm^2^
**Medium**	MEM-α
**CO_2_ concentration**	5.0%
**Temperature**	37.0 ℃
**Incubation time**	72 h
**pH**	6.8–7.2

**Table 3:** Optical constants, film thickness, and amorphous carbon film type obtained using spectroscopic ellipsometry.

Sample No.	χ^2^	Film Thickness (nm)	n	k	Amorphous Carbon Type
			λ = 550 nm		
1	0.10	108	1.930	0.040	PLC (not DLC)
2	0.02	150	2.225	0.258	a-C:H (DLC)
3	0.05	90	2.500	0.268	ta-C:H (DLC)
4	0.04	175	1.870	0.415	GLC (not DLC)
5	0.03	120	2.290	0.690	a-C (DLC)

DLC, diamond-like carbon; PLC, polymer-like carbon; a-C, amorphous carbon; a-C:H, hydrogenated amorphous carbon; ta-C:H, hydrogenated tetrahedral amorphous carbon; GLC, graphite-like carbon.

**Table 4:** Gaussian fitting results of Raman spectra.

Sample No.	Amorphous Carbon Type	UV Sterilization at 253.7 nm	D-Peak Position (cm^−1^)	G-Peak Position (cm^−1^)	FWHM (G)	I_D_/I_G_ Intensity Ratio
1	PLC	Without UV	1305 ± 1.3	1523 ± 0.3	165.2 ± 0.5	0.31
		With UV	1311 ± 1.6	1524 ± 0.3	162.4 ± 0.5	0.32
2	a-C:H	Without UV	1361 ± 2.0	1541 ± 0.2	175.8 ± 0.7	0.52
		With UV	1355 ± 2.1	1539 ± 0.2	173.9 ± 0.7	0.52
3	ta-C:H	Without UV	1378 ± 2.6	1553 ± 0.3	176.6 ± 1.0	0.61
		With UV	1377 ± 2.6	1552 ± 0.2	174.2 ± 1.1	0.63
4	GLC	Without UV	1384 ± 0.5	1584 ± 0.1	127.0 ± 0.5	1.00
		With UV	1383 ± 0.5	1584 ± 0.1	127.1 ± 0.5	0.98
5	a-C	Without UV	1395 ± 0.7	1579 ± 0.2	132.8 ± 0.8	1.35
		With UV	1389 ± 0.8	1577 ± 0.2	137.2 ± 0.9	1.27

PLC, polymer-like carbon; a-C, amorphous carbon; a-C:H, hydrogenated amorphous carbon; ta-C:H, hydrogenated tetrahedral amorphous carbon; GLC, graphite-like carbon.

**Table 5:** Wettability and surface roughness of amorphous carbon films classified based on optical constants.

Sample No.	Amorphous Carbon Type	UV Sterilization at 253.7 nm	Pure Water Contact Angle (Degree) (n = 10)	AFM
				Surface Roughness (nm)
1	PLC	Without UV	71 ± 4	1.2 ± 0.7 (n = 4)
		With UV	70 ± 3	2.5 ± 2.0 (n = 4)
2	a-C:H	Without UV	71 ± 5	1.1 ± 0.1 (n = 3)
		With UV	68 ± 1	2.0 ± 0.1 (n = 3)
3	ta-C:H	Without UV	65 ± 3	1.4 ± 0.1 (n = 3)
		With UV	63 ± 2	2.1 ± 0.1 (n = 3)
4	GLC	Without UV	78 ± 2	2.1 ± 0.0 (n = 3)
		With UV	75 ± 4	2.0 ± 0.2 (n = 3)
5	a-C	Without UV	77 ± 5	2.2 ± 0.2 (n = 3)
		With UV	71 ± 5	2.1 ± 0.1 (n = 3)
Control (Si)	-	Without UV	-	0.5 ± 0.4 (n = 3)
		With UV	-	0.5 ± 0.2 (n = 3)

Data are presented as mean ± standard deviation. AFM, atomic force microscopy; PLC, polymer-like carbon; a-C, amorphous carbon; a-C:H, hydrogenated amorphous carbon; ta-C:H, hydrogenated tetrahedral amorphous carbon; GLC, graphite-like carbon.

**Table 6:** Surface composition of amorphous carbon films classified based on optical constants.

Sample No.	Amorphous Carbon Type	UV Sterilization at 253.7 nm	O1s/C1s Ratio	C1s Curve Fitting Area				
				C-C sp^2^	C-C sp^3^	C-O	C=O	O=C-O
1	PLC	Without UV	0.14	0.92	0.22	0.04	0.02	0.00
		With UV	0.16	0.95	0.20	0.05	0.02	0.00
2	a-C:H	Without UV	0.13	0.90	0.29	0.06	0.03	0.00
		With UV	0.19	0.86	0.20	0.07	0.03	0.01
3	ta-C:H	Without UV	0.14	0.95	0.12	0.06	0.05	0.01
		With UV	0.16	0.93	0.22	0.05	0.04	0.00
4	GLC	Without UV	0.35	0.87	0.28	0.14	0.11	0.03
		With UV	0.39	0.87	0.27	0.16	0.12	0.03
5	a-C	Without UV	0.27	0.90	0.18	0.10	0.08	0.01
		With UV	0.30	0.91	0.19	0.11	0.08	0.02

PLC, polymer-like carbon; a-C, amorphous carbon; a-C:H, hydrogenated amorphous carbon; ta-C:H, hydrogenated tetrahedral amorphous carbon; GLC, graphite-like carbon.

**Table 7:** Osteoblast proliferation of amorphous carbon films classified based on optical constants.

Sample No.	Amorphous Carbon Type	Osteoblast Proliferation (n = 6 × 5 Cycles) ^1^	*p*-Value for Control
1	PLC	1.1 ± 0.3	*p* > 0.05 (control level)
2	a-C:H	1.0 ± 0.2	*p* > 0.05 (control level)
3	ta-C:H	0.9 ± 0.1	*p* > 0.05 (control level)
4	GLC	1.5 ± 0.3	*p* < 0.01 (high)
5	a-C	1.1 ± 0.2	*p* > 0.05 (control level)
Control (Si)	-	1.0 ± 0.1	-

PLC, polymer-like carbon; a-C, amorphous carbon; a-C:H, hydrogenated amorphous carbon; ta-C:H, hydrogenated tetrahedral amorphous carbon; GLC, graphite-like carbon. ^1^ Data are presented as mean ± standard deviation.

## Data Availability

The data presented in this study are available on request from the corresponding author.
